# Probing Coagulation Behavior of Individual Aluminum Species for Removing Corresponding Disinfection Byproduct Precursors: The Role of Specific Ultraviolet Absorbance

**DOI:** 10.1371/journal.pone.0148020

**Published:** 2016-01-29

**Authors:** He Zhao, Chengzhi Hu, Di Zhang, Huijuan Liu, Jiuhui Qu

**Affiliations:** 1 State key Laboratory of Environmental Aquatic Chemistry, Research Center for Eco-Environmental Sciences, Chinese Academy of Sciences, Beijing, 100085, China; 2 Beijing Engineering Research Center of Process Pollution Control, Key Laboratory of Green Process and Engineering, Institute of Process Engineering, Chinese Academy of Sciences, Beijing, 100190, China; NSYSU, TAIWAN

## Abstract

Coagulation behavior of aluminum chloride and polyaluminum chloride (PACl) for removing corresponding disinfection byproduct (DBP) precursors was discussed in this paper. CHCl_3_, bromine trihalomethanes (THM-Br), dichloroacetic acid (DCAA) and trichloroacetic acid (TCAA) formation potential yields were correlated with specific ultraviolet absorbance (SUVA) values in different molecular weight (MW) fractions of humic substances (HS), respectively. Correlation analyses and principal component analysis were performed to examine the relationships between SUVA and different DBP precursors. To acquire more structural characters of DBP precursors and aluminum speciation, freeze-dried precipitates were analyzed by fourier transform infrared (FTIR) and C 1s, Al 2p X-ray photoelectron spectroscopy (XPS). The results indicated that TCAA precursors (no MW limits), DCAA and CHCl_3_ precursors in low MW fractions (MW<30 kDa) had a relatively good relations with SUVA values. These DBP precursors were coagulated more easily by *in situ* Al_13_ of AlCl_3_ at pH 5.0. Due to relatively low aromatic content and more aliphatic structures, THM-Br precursors (no MW limits) and CHCl_3_ precursors in high MW fractions (MW>30 kDa) were preferentially removed by PACl coagulation with preformed Al_13_ species at pH 5.0. Additionally, for DCAA precursors in high MW fractions (MW>30 kDa) with relatively low aromatic content and more carboxylic structures, the greatest removal occurred at pH 6.0 through PACl coagulation with aggregated Al_13_ species.

## Introduction

Humic substances (HS), which widely exists in raw water, can react with chlorine in water treatment process to form halogenated disinfection byproducts (DBPs) [1]. For DBPs control, enhanced coagulation is considered to be one of the best available techniques (BATs) [2, 3]. Aluminum salts, such as aluminum sulfate, aluminum chloride (AlCl_3_) and polyaluminum chloride (PACl) coagulants, are commonly used for reducing DBP precursors in drinking water treatment [4–8]. Previous investigations demonstrated aluminum species showed *in situ* Al_13_ species of AlCl_3_ at pH 5.0 (or aggregated Al_13_ of PACl at pH 6.0) were preferentially bound to DBP precursors with aromatic and carboxylic structures [7]. Other studies reported that Al_13_ species selectively bound to carboxylic groups at pH 6.0, and to phenolic moieties at pH 8.0 [9].

On the other hand, extensive researchers have found that trihalomethanes (THMs) and haloacetic acids (HAAs) formation is strongly dependent on the characteristics of DBP precursors [10, 11]. Specific ultraviolet absorbance (SUVA) value is a good surrogate for aromatic content of organic matter [12, 13]. It has been reported that SUVA influences significantly on DBPs reactivity [10, 11, 14]. Many efforts have been made to correlate molecular weight (MW) or structure of DBP precursors to individual DBPs formation potential (DBPsFP) [14–23] using HPSEC and XAD method [13, 24–27]. Some studies have concluded that dichloroacetic acid (DCAA) and trichloroacetic acid (TCAA) have different precursors, also form through distinct pathways [16, 17]. Further research reported that hydrophilic and low molecular weight (<0.5 kDa) fractions gave the highest contribution for dihalogenated HAA yields [17].

Recently, the role of aluminum speciation in the coagulation has attracted more attention. Our previous study indicated both structure characterization of DBP precursors and aluminum speciation could affect coagulation. However, few associate the coagulation behavior of individual aluminum species with corresponding DBP using SUVA. Furthermore, the role of SUVA as an indicator of aromaticity in coagulation behavior still needs further investigation.

The primary objective of the present research was to probe coagulation behavior of aluminum species for removing corresponding DBP precursors. DBPs formation potential (DBPsFP) in different MW fractions (including CHCl_3_FP/DOC, THMFP-Br/DOC, DCAAFP/DOC and TCAAFP/DOC) were correlated with specific ultraviolet absorbance (SUVA) values. Correlation analyses (CA) and principal component analysis (PCA) were performed to examine the relationships between SUVA and different DBP precursors. To acquire more structural distribution, freeze-dried raw waters and flocs by AlCl_3_ and PACl coagulation were analyzed by fourier transform infrared (FTIR). We further identified surface component of organics and aluminum in the flocs by C 1s and Al 2p X-ray photoelectron spectroscopy (XPS).

## Experimental Methods

### Jar Tests

The HS was extracted from the sediments of Hanshiqiao Wetland in Beijing, China. The elemental composition of C, H, N and O was 31.17, 4.07, 3.56, and 30.34 wt %, respectively. Fractionation of HS was performed on a stirred ultrafiltration cell device (Model 8200, Amicon, Millipore, USA) with nominal MW cutoffs of 3, 10, 30, 100, 300 kDa regenerated cellulose membranes (PL, 63.5mm, Millipore, USA). Details were in accordance with previous study [6]. Raw HS and each fraction were first diluted in 1 L of deionized water to form a DOC concentration of 4.83 mg/L (±0.06 mg/L). Sodium bicarbonate was added to produce a final alkalinity of 100 mg/L as CaCO_3_, potassium chloride was added to bring the ionic strength to 3.3 mmol/L, and kaolinite was added to produce an approximate turbidity of 20 NTU. This solution was mixed for 2 h, and then left in a closed container overnight before it was used in jar tests. This interval allowed the clay material to equilibrate with the water.

Jar tests were performed on a programmable jar tester (MY3000-6, MeiYu, China) in 500 mL beakers at room temperature. After the coagulants were injected into the HA samples, 2 min of rapid mixing at 200 rpm, and 15 min of slow stirring at 40 rpm was provided, followed by 30 min of quiescent settling. The pH of solutions was adjusted during rapid mixing.

AlCl_3_·6H_2_O (Guaranteed Reagent, Beijing Chemical Regents Company) and PACl with high Al_13_ content (obtained from Prof. Baoyou Shi in Research Center for Eco-Environmental Sciences, Chinese Academy of Sciences [28]) were used for all jar tests. The basicity value (OH/Al molar ratio) of PACl is 2.1, and the content of Al_13_ is 81.2%. After supernatants through 0.45 μm cellulose acetate membrane filters for testing DOC, UV_254_ and DBPsFP, precipitates were freeze-dried for infrared and XPS analysis.

### DBPs formation potential

Prior to the addition of chlorine, all water samples were adjusted to pH 7.0 ± 0.2. Buffer solution of NaOH/KH_2_PO_4_ (pH 7.0) and HOCl stock solution (an applied chlorine dosage of 20 mg/L) were injected into each water sample, and then it was capped and put in a 20°C incubator. After the 72-hour incubation, residual chlorine in the water samples was measured by the DPD titrimetric method. All samples were found to have measurable free chlorine residual. Water samples were then added with sodium sulfite and analyzed to determine THMFP and HAAFP concentrations.

Four THMs (CHCl_3_, CHBrCl_2_, CHBr_2_Cl and CHBr_3_), dichloroacetic acid and trichloroacetic acid were measured following the U.S. EPA method 551 and 552.3 [29], respectively. Quantitive analysis was conducted using a gas chromatograph (6890N, Agilent, USA) with an electron capture detector (ECD).

THMs samples were extracted with hexane (HPLC Grade, Fisher, USA), and HAAs samples were extracted with methyl-tert-butyl ether (MTBE) (HPLC Grade, J.T. Baker, USA) followed by derivation with acidic methanol. 1, 2-dibromopropane (≥98.0%, GC, Fluka, USA) served as the interval standard. Conditions for the analyses were as follows: (1) THMs, injector temperature 200°C, column temperature 35°C (holding 4 min) to 260°C (10°C/min), detector temperature 290°C; (2) HAAs, injector temperature 200°C, column temperature 35°C (holding 4 min) to 65°C (2°C/min), detector temperature 290°C.

### Characterization of flocs

The infrared spectra were obtained on a Nicolet 5700 FTIR spectrometer (Thermo Electron Corporation, U.S.A.), using 2-5mg of flocs in potassium bromide pellets. XPS analysis was performed on an X-ray photoelectron spectrometer (ESCALAB 250, Thermo VG Scientific, UK) with a monochromatized Al Kα X-ray source (1486.7 eV) working at 150 W and 15 kV. High resolution scans were conducted with pass energy of 20 eV and step size of 0.1 eV.

### Statistical analyses

CA and PCA were performed with SPSS 13.0 software. PCA was conducted using the relative abundance of DBP precursors in different MW fractions by two principal components analyses. CA was used to examine the relationships between SUVA and different DBP precursors. Significance levels are reported as non-significant (NS) (p > 0.05), significant (*, 0.05 > p > 0.01) or highly significant (**, p < 0.01).

## Results and Discussion

### DBPsFP in different MW fractions before and after coagulation

Specific CHCl_3_FP, THMFP-Br, DCAAFP and TCAAFP yields in the individual MW fractions of raw waters are presented in Table 1. High MW fraction (>30 kDa) produced lower CHCl_3_FP, THMFP-Br, DCAAFP and TCAAFP yields, whereas 3-10k Da and <3 kDa fractions produced higher DBPsFP. The general trend was that lower MW fractions had more reactive DBPs precursors, especially for TCAA precursors. Organic matter in low MW with higher reactivity of DBPs formation was also observed by other researchers [14, 15]. In contrast with THMsFP yields, DCAA and TCAA yields had higher formation potential. That is, DCAA and TCAA precursors were main DBPs precursors in each fraction. It was consistent with the finding that the relative concentration of HAAs usually was greater than THMs in high-SUVA waters [16, 30].

**Table 1:** Individual DBPsFP specific yields (μg/mg C) in different MW fractions of raw and coagulated HS waters.

DBPsFP		raw water	AlCl_3_ ^*a*^		PACl ^*a*^	
			pH 5.0	pH 6.0	pH 5.0	pH 6.0
**CHCl**_**3**_**FP**	**>100k Da**	23.2±0.8	15.1±1.5	18.4±3.1	13.7±0.1	17.9±1.9
**CHCl**_**3**_**FP**	**30-100k Da**	27.0±1.8	15.2±0.1	24.1±1.3	14.2±1.0	17.4±0.2
**CHCl**_**3**_**FP**	**10-30k Da**	53.8±3.0	17.7±1.5	24.9±1.1	18.7±0.7	15.9±1.1
**CHCl**_**3**_**FP**	**3-10k Da**	57.4±7.5	25.2±0.3	28.2±1.4	27.6±2.2	29.8±0.5
**CHCl**_**3**_**FP**	**<3k Da**	57.3±2.8	27.3±0.1	45.8±5.4	27.8±1.2	28.7±0.9
**THMFP-Br**	**>100k Da**	8.8±0.7	21.0±0.2	16.3±0.3	14.9±0.1	16.6±0.7
**THMFP-Br**	**30-100k Da**	9.6±1.3	23.5±0.2	15.2±2.5	12.2±0.1	17.7±0.1
**THMFP-Br**	**10-30k Da**	14.4±0.4	15.3±0.4	10.6±1.1	9.1±0.1	11.2±0.9
**THMFP-Br**	**3-10k Da**	13.1±1.5	8.1±0.2	12.8±1.1	5.5±0.3	13.0±1.2
**THMFP-Br**	**<3k Da**	13.1±0.2	8.8±0.1	13.9±2.7	7.9±0.3	8.1±0.3
**DCAAFP**	**>100k Da**	49.7±1.5	36.3±2.0	35.5±2.4	41.5±5.8	33.0±0.5
**DCAAFP**	**30-100k Da**	51.7±1.4	37.2±1.9	39.4±3.2	41.3±3.3	29.0±1.9
**DCAAFP**	**10-30k Da**	65.5±2.3	39.3±0.3	46.3±3.2	30.6±7.3	34.4±0.3
**DCAAFP**	**3-10k Da**	79.8±6.8	42.3±1.9	50.7±6.3	49.6±2.6	44.5±1.8
**DCAAFP**	**<3k Da**	88.9±5.0	55.4±0.6	98.2±8.1	63.4±2.2	76.7±2.1
**TCAAFP**	**>100k Da**	38.8±1.8	16.2±0.2	27.1±0.4	24.9±4.8	16.6±0.6
**TCAAFP**	**30-100k Da**	32.5±1.9	14.2±1.3	22.3±2.7	21.3±3.2	19.5±3.7
**TCAAFP**	**10-30k Da**	52.0±6.1	13.9±0.3	35.7±5.6	19.0±1.2	16.2±0.5
**TCAAFP**	**3-10k Da**	68.4±10.7	23.2±1.9	54.1±3.1	64.6±7.4	26.1±0.9
**TCAAFP**	**<3k Da**	107.2±8.3	38.9±2.5	101.6±9.9	81.7±5.7	58.7±3.0

^*a*^ Coagulants dose = 0.8 mg Al/mg DOC.

Table 1 also summarizes the residual CHCl_3_FP, THMFP-Br, DCAAFP and TCAAFP specific yields in individual MW fractions after coagulation, respectively. Lower DBPsFP yields suggested greater removal efficiencies of DBP precursors by AlCl_3_ and PACl coagulation. The greatest removal of specific TCAAFP yields occurred at pH 5.0 by AlCl_3_ coagulation in all MW fractions. In contrast to TCAA precursors, AlCl_3_ and PACl coagulation were not responsible for reducing the THM-Br precursors, especially for high MW fractions. Comparing with the original yields, residual THMFP-Br yields in >100k Da and 30-100k Da fractions all increased after coagulation. The specific THMFP-Br yields reached minimum at pH 5.0 by PACl coagulation. Increased THMFP-Br yields may be attributed to worse removals for these DBP precursors than average DOC removal by coagulation. For DCAA and CHCl_3_ precursors, removals by coagulation were significantly different in high or low MW fractions. For DCAA precursors in low MW fractions (MW<30 kDa), the greatest removal was AlCl_3_ coagulation at pH 5.0. In high MW fraction (>100 kDa and 30–100 kDa), the residual DCAAFP yields reached minimum (33.0 and 29.0 μg/mg DOC, respectively) at pH 5.0 by PACl coagulation. In contrast to DCAA precursors, the greatest removals of CHCl_3_ precursors by coagulation were different in high MW fractions, but similar with DCAA precursors in low MW fractions. In high MW fractions (MW>30 kDa), the greatest removal of CHCl_3_ precursors by coagulation was with PACl at pH 5.0. In low MW fractions (MW<30 kDa), the greatest removal was AlCl_3_ coagulation at pH 5.0.

### Correlations between SUVA values and individual DBPsFP in different MW fractions

**Fig 1:**
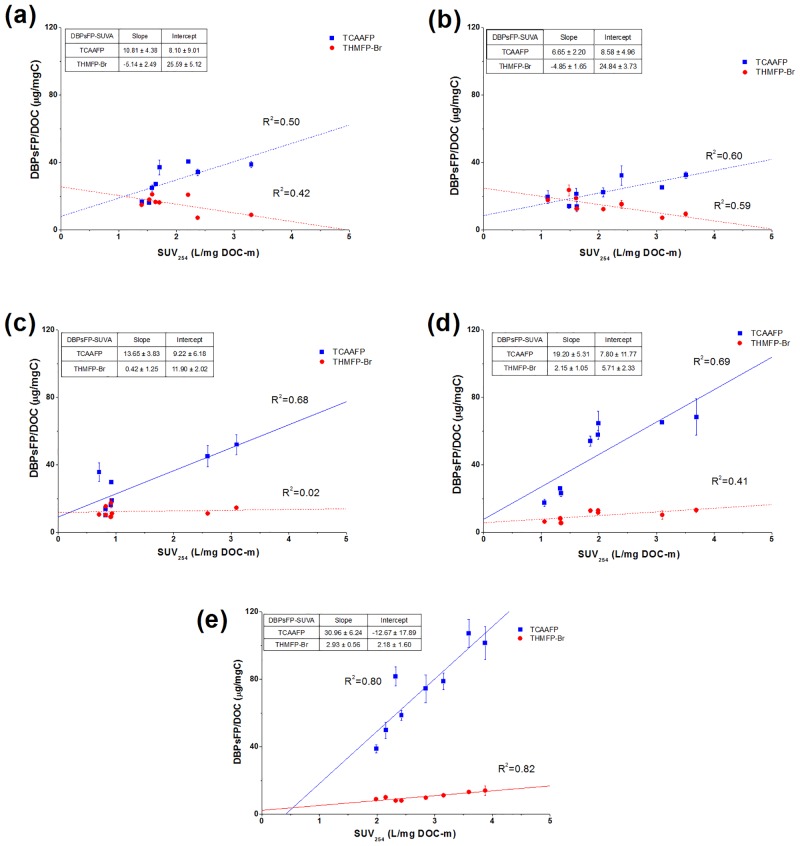
Correlations of TCAAFP and THMFP-Br yields with SUVA values in the raw and coagulated waters. (a) >100k Da fraction; (b) 30-100k Da fraction; (c) 10-30k Da fraction; (d) 3-10k Da fraction; (c) <3k Da fraction.

**Fig 2:**
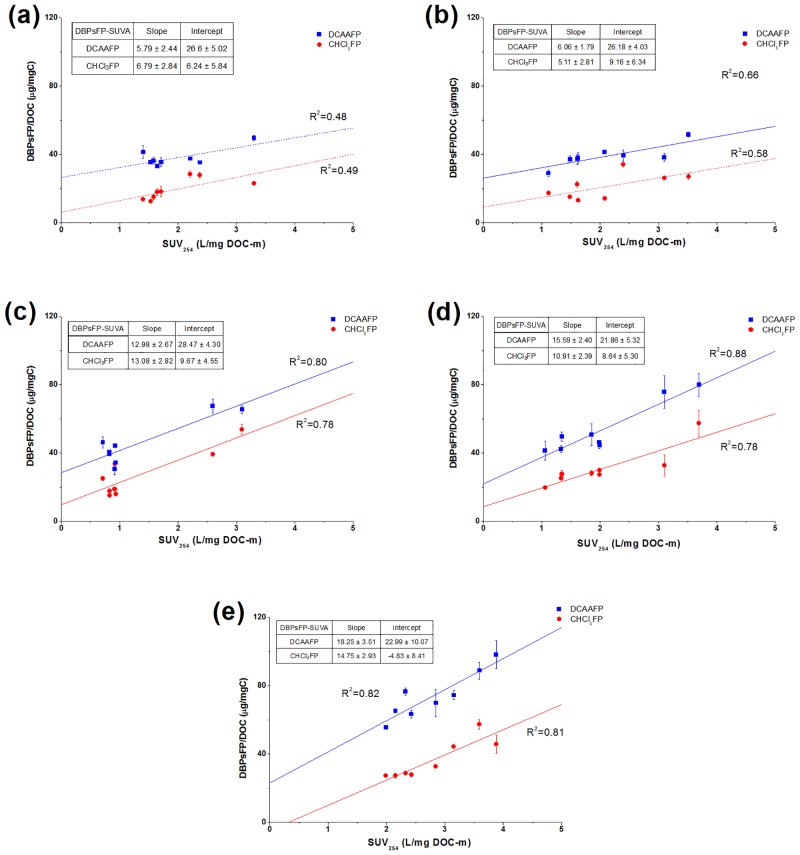
Correlations of DCAAFP and CHCl_3_FP yields with SUVA values in the raw and coagulated waters. (a) >100k Da fraction; (b) 30-100k Da fraction; (c) 10-30k Da fraction; (d) 3-10k Da fraction; (c) <3k Da fraction.

SUVA is a good surrogate for aromatic content of organic matter. It has been reported that SUVA influences significantly on DBPs reactivity [11, 15, 31]. In this study, correlations of TCAAFP, THMFP-Br and DCAAFP, CHCl_3_FP yields with SUVA values in different MW fractions (including raw and coagulated waters) were investigated (Figs 1 and 2). Spearman’s correlation coefficients (R) were calculated (Table 2 and S1 Table in S1 File). Among the qualitative parameters, there was a significant correlation between SUVA and TCAAFP, CHCl_3_FP, DCAAFP yields (*R* = 0.727, 0.722, and 0.732, respectively. *p* < 0.01). In the regression lines, correlation coefficient (R^2^) and slope were parameters for contribution of SUVA on DBPsFP, and intercept was a parameter for contribution of other function groups (e.g., carboxyl) for DBPsFP. As illustrated in Figs 1 and 2, the slopes and R^2^ of correlations mostly increased with MW decreased. Thus, SUVA provided more contribution on DBPsFP in low MW fractions.

**Table 2:** Correlation matrix of SUVA index and four DBPsFP.

	SUVA	TCAAFP	THMFPBr	CHCl_3_FP	DCAAFP
**SUVA**	1				
**TCAAFP**	.727**	1			
**THMFPBr**	-.177	-.172	1		
**CHCl**_**3**_**FP**	.722**	.788**	-.142	1	
**DCAAFP**	.732**	.886**	-.270	.798**	1

** Correlation is significant at the 0.01 level (2-tailed).

The SUVA values showed positive correlations with the TCAAFP yields in all MW fractions (Fig 1). SUVA-TCAAFP correlations had higher slopes and correlation coefficients, but lower intercepts in low MW fractions. SUVA is a good indicator of aromatic carbon contents, and higher SUVA values represent stronger aromaticity [31]. In this study, positive linear relationships and high slope of the SUVA-TCAAFP relations confirmed that aromatic carbon sites gave great contribution to TCAAFP in low MW fractions. S1 Table in S1 File also showed a significant correlation between SUVA and TCAAFP yields in >100 kDa, 30–100 kDa, 10–30 kDa and <10 kDa fractions (*R* = 0.710, 0.777, 0.824 and 0.828, respectively. *p* < 0.05).

However, in contrast to SUVA-TCAAFP, the correlations of SUVA and THMFP-Br were different. In high MW fractions (MW>30k Da), the SUVA values showed negative correlations with the yields of THMFP-Br in MW>100 and 30-100k Da fractions (*R* = -0.645 and -0.767, respectively. *p* < 0.05). Though the correlation coefficients of SUVA-THMFP-Br in low MW fractions were positive, the low slopes indicated that aromatic structures only did a little contribution to the THMFP-Br formation, especially in the 10-30k Da fraction. There was no obvious correlation between the SUVA and the THMFP-Br yields in the 10-30k Da fraction (*R* = 0.0135). The negative relationships in high MW fractions were explained by some researchers [16] that certain functional groups in DBP precursors may affect the formation of THM-Br. This result also compares favorably with the observations that bromine was more reactive with aliphatic precursors than with aromatic precursors, and the reverse was true for chlorine [32]. Through negative relationships between SUVA and THMFP-Br in high MW fractions, it can be concluded that aromatic structures with high MW were no contribution to THMFP-Br, but other structures (e.g., aliphatics) may be a contributor.

As present in Fig 2, the SUVA values also correlated with both DCAAFP yields and CHCl_3_FP yields in all MW fractions. The correlations of SUVA-DCAAFP and SUVA-CHCl_3_FP were good linear relations with higher slopes and correlation coefficients in low MW fractions (MW<30k Da), but weak linear relationships in high MW fractions (MW>30k Da) were observed between the SUVA and the yields of DCAAFP (or CHCl_3_FP) with low slopes and R^2^. Significant correlations between SUVA and DCAAFP and CHCl_3_FP yields was shown in low MW fractions (*R* = 0.884 and 0.893 in 10–30 kDa fractions, and *R* = 0.881 and 0.936 in <10 kDa fractions respectively. *p* < 0.01). However, the correlations between SUVA and DCAAFP and CHCl_3_FP yields in high MW fractions were not significant (*R* = 0.699 and 0.696 in >100 kDa fractions, and *R* = 0.636 and 0810 in 30–100 kDa fractions respectively. *p* < 0.05). The trends of increasing DCAA (or CHCl_3_) formation potential with increasing SUVA values were clear. However, the slopes of SUVA-DCAAFP (or SUVA-CHCl_3_FP) relations were not as high as those of SUVA-TCAAFP relations. That is, although aromatic structures provided some contribution to the DCAAFP (or CHCl_3_FP), more aromatic structures contributed on the TCAAFP, especially in low MW fractions. These results were consistent with the observations by Liang and Singer [16]. Their results indicated that TCAA precursors are relatively more hydrophobic than DCAA and THM precursors. Furthermore, comparing with the correlations of SUVA-CHCl_3_FP, higher intercepts of the SUVA-DCAAFP relations indicated that other functional groups (e.g., carboxyl) provided more contribution for DCAAFP. Some researchers presented that DCAA and TCAA species had different formation mechanisms or different precursors, while THMs may have relatively more aliphatic moieties as their precursors in addition to aromatic structures [1, 16]. However, in this study, it should be noted that activated aromatic structures are still an important part for DCAA and CHCl_3_ precursors, especially in low MW fractions. It can be inferred that different coagulation efficiencies for removing individual DBPsFP (Table 1) may be attributed to structural characteristics of DBPs precursors.

### Analysis of SUVA values and individual DBPsFP in different MW fractions by PCA

**Fig 3:**
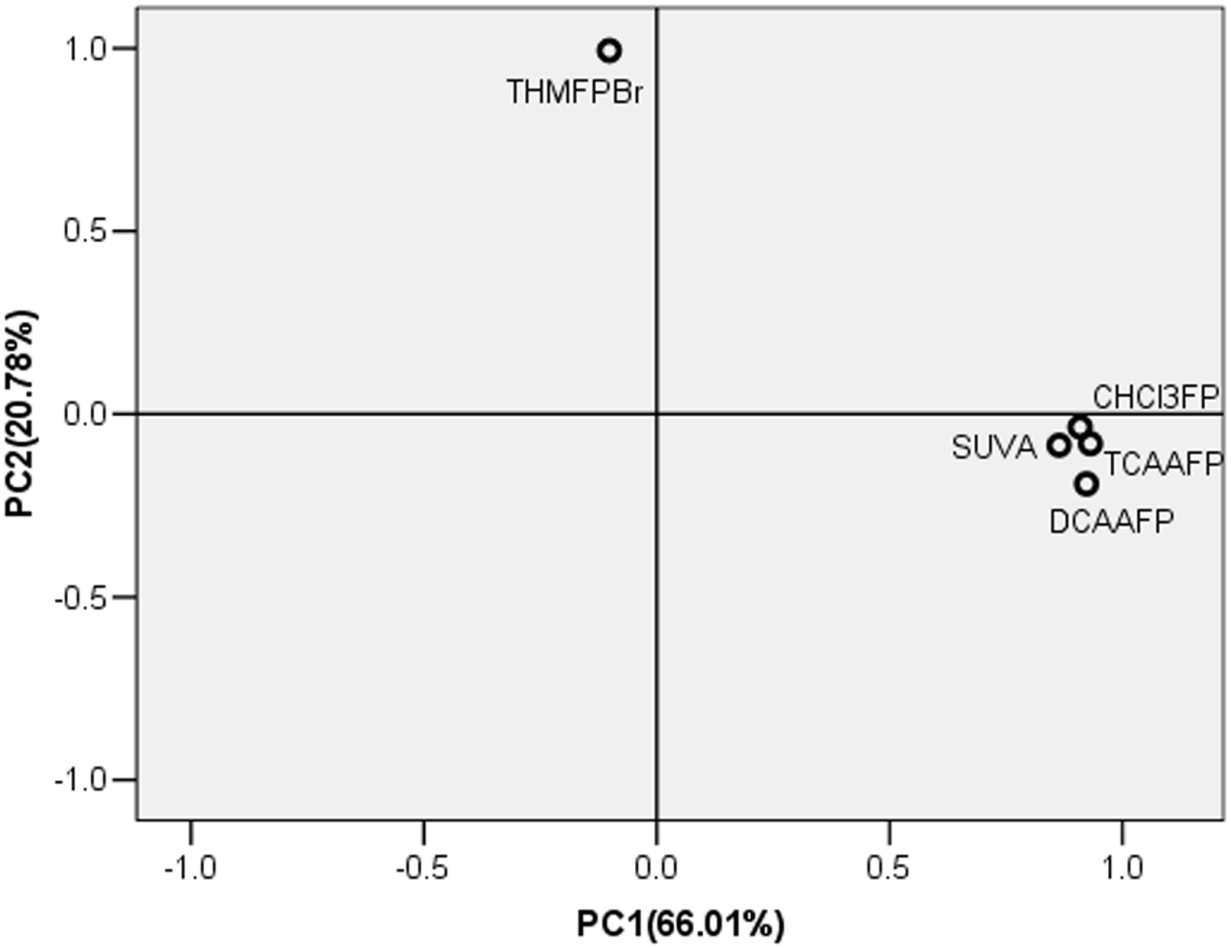
Property—property plots of PCA factor loadings between four individual DBPsFP and SUVA index.

PCA was conducted by the specific yields of four individual DBPsFP and SUVA index. The result is showed in Fig 3 and S1 Fig in S1 File. For all samples in Fig 3, factor 1 and factor 2 of PCA accounted for 66.01% and 20.78%, respectively. The summation of the two principal components has already accounted for 86.79%, enough to explain the whole variation tendency of parameters. Each PCA factor was a linear combination of four parameters where the measured factors are dimensionless and can be either positive or negative:

$$\text{F}1\;=\;0.86\text{ SUVA}+0.93\text{ TCAAFP}-0.29\text{ THMFPBr}+0.90\text{ CHC}{\text{l}}_{3}\text{FP}+0.94\text{ DCAAFP}$$ (1)

$$\text{F}2\;=\;0.08\text{ SUVA}+0.1\text{ TCAAFP}+0.96\text{ THMFPBr}+0.14\text{ CHC}{\text{l}}_{3}\text{FP }-0.01\text{ DCAAFP}$$ (2)

Fig 3 showed the property—property plots between first and second factor loadings. All the parameters except THMFPBr showed positive factor 1 loadings. Wherein, the THMFPBr component was less significant related to factor 1 axe than the other four parameters, while showed highly positive factor 2 loadings. The other four parameters (SUVA, TCAAFP, CHCl_3_FP and DCAAFP) were close to the factor 1 axis and neared zero for the factor 2 loadings. The PCA results indicated that TCAAFP, CHCl_3_FP and DCAAFP maybe had close relations with SUVA dominated by factor 1. Hence, the PCA in our present study could separate the characteristics of the SUVA with different DBPsFP.

**Fig 4:**
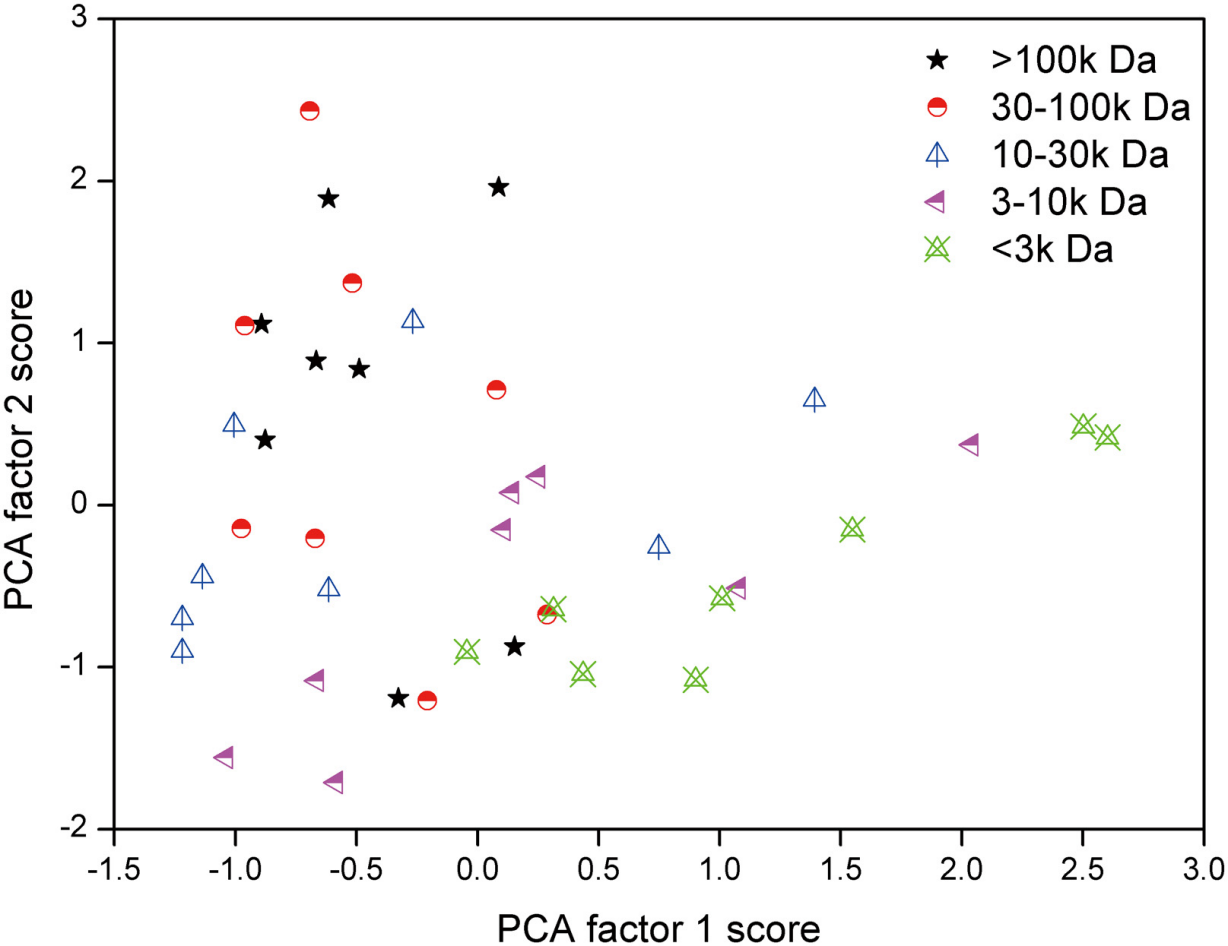
PCA factor scores of all the samples classified by different MW. >100k Da fraction; 30-100k Da fraction; 10-30k Da fraction; 3-10k Da fraction; <3k Da fraction.

The PCA factors 1 and 2 scores of all 40 samples are plotted in Fig 4. The figure showed a marked difference between individual MW samples. The scores of different MW samples were relatively scattered. This result indicated that the effect of SUVA to DBPsFP was variable by different MWs. In general, with the decreasing of MWs, the factor 2 scores of DBPsFP samples decreased accompanied with the increasing of factor 1 scores.

Most lower MWs samples clustered with higher factor 1 and lower factor 2 scores, with MW <3k Da samples ranging from -0.04 to 2.6 for PCA 1 and -1.08 to 0.48 for PCA 2, MW 3-10k Da samples ranging from -0.66 to 2.04 for PCA 1 and -1.71 to 0.37 for PCA 2, respectively. In contrast, most of the higher MWs samples were located in the regions with lower factor 1 and higher factor 2 scores (approximate -0.97 to 0.28 for PCA 1 and -1.2 to 2.43 for PCA 2 in MW 30-100kDa fractions, -0.89 to 0.15 for PCA 1 and -1.19 to 1.96 for PCA 2 in MW >100kDa fractions, respectively). While there was no apparent pattern for most of the MW 10-30kDa fractions with ranging from -1.22 to 1.40 for PCA 1 and -0.90 to 1.13 for PCA 2. Obviously, it can be identified that DBPsFP in lower MWs was mainly influenced by factor 1, while DBPsFP in higher MWs was mainly dominated by factor 2. Therefore, lower MW fractions were more relevant to SUVA index in our study.

### Characterization of raw waters and flocs in MW fractions by FTIR

**Fig 5:**
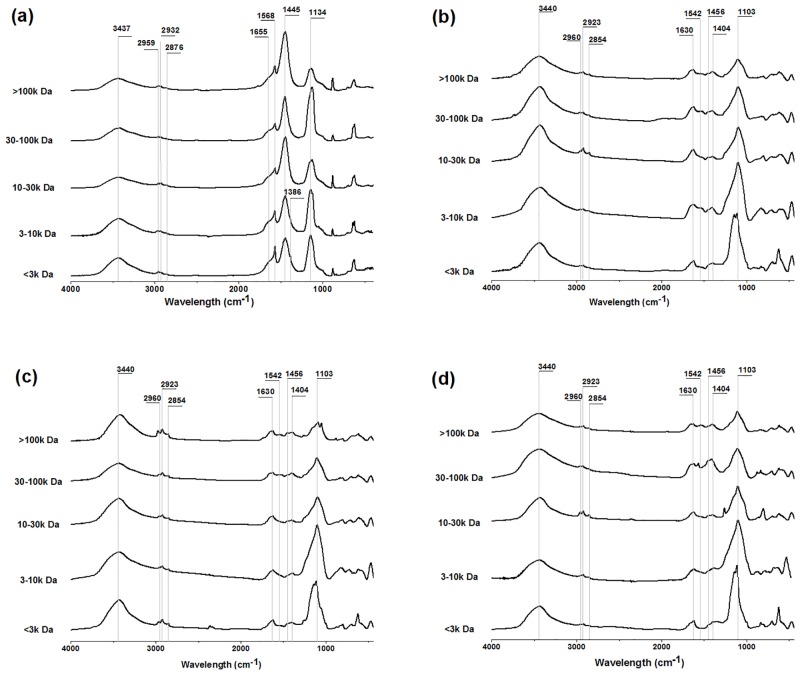
FTIR spectra of raw waters and freeze-dried flocs by coagulation in different MW fractions. (a) raw waters; (b) AlCl_3_, pH 5.0; (c) PACl, pH 5.0; (d) PACl, pH 6.0. Coagulants dose: 0.8 mg Al/mg DOC.

As illustrated in Fig 5a, FTIR spectra of raw waters indicated significant differences among the MW fractions. The MW>100k Da fraction showed very strong absorption at 1445 cm^-1^. Pronounced absorbance at 1440–1460 cm^-1^, which was assigned as C—H deformation vibration of aliphatic structure [33, 34], became progressively weaker with a decrease in MW. It indicated an increase in aliphatic content with increasing MW. A point of interest was that peaks at 1655 and 1568 cm^-1^ were inversely related to the intensity of absorption in the 1445 cm^-1^. The peak at 1620–1660 cm^-1^ and 1540–1570 cm^-1^ mainly due to aromatic C = C stretching (or C = O stretching of conjugated carbonyl groups) and C = O stretching vibration of ketones/quinones [33–35]. The intensities of bands in the 1655 and 1568 cm^-1^ were stronger in low MW fractions than those in high MW fractions, indicating higher contents of aromatic structures and carbonyl groups in low MW fractions. Additionally, shoulder peaks appeared at near 1386 cm^-1^ in <3k Da and 3-10k Da fractions also indicated higher carboxylic or other oxygen-containing groups in low MW fractions. This shoulder peak was commonly assigned to O-H deformation and C-O stretching of phenolic or carboxylic group [33, 35]. Accordingly, it suggested that high MW fractions had more aliphatic carbon structures and low MW fractions had higher contents of aromatic structures and oxygen-containing groups. It was consistent with the results of relationships between SUVA and DBPsFP yields in different MW fractions (Figs 1–4) discussed above. Due to more aromatic structures and less aliphatic structures in low MW fractions, almost all SUVA-DBPsFP relations in MW<30k Da fractions had higher slopes and correlation coefficients (Figs 1 and 2).

FTIR analysis of the coagulation precipitates are presented in Fig 5 and Table 3. The region of 1440–1460 cm^-1^ was greatly reduced in intensity by coagulation. The peak at 1655 cm^-1^ corresponding to aromatic structures [33, 35] shifted to lower frequency with higher energy (1620–1640 cm^-1^) and increased in intensity. 1630/2923 (1630/2854) values indicated the ratios of aromatic to aliphatic structures. The 1630/2923 (1630/2854) ratios of flocs reached maximum at pH 5.0 by AlCl_3_ coagulation, while that achieved minimum mostly at pH 5.0 by PACl coagulation in all MW fractions. These results demonstrated the trends of aromatic and aliphatic moieties in the flocs under different coagulation conditions. This was consistent with the hypothesis proposed above (Table 1 and Figs 1 and 2) that different coagulation removal of individual DBPsFP was due to structural characteristics of DBPs precursors.

**Table 3:** Characterization for coagulation flocs by FTIR.

FTIR data ^*a*^			>100k Da	30-100k Da	10-30k Da	3-10k Da	<3k Da
**AlCl**_**3**_ **pH 5.0** ^*b*^	**Aromatics /aliphatics**	1630/2923	1.30	1.68	0.90	1.62	1.71
**AlCl**_**3**_ **pH 5.0** ^*b*^	**Aromatics /aliphatics**	1630/2854	2.01	2.65	1.42	2.04	2.52
**AlCl**_**3**_ **pH 5.0** ^*b*^	**C = O/ aliphatics**	1542/2923	0.77	1.15	0.37	1.14	0.81
**AlCl**_**3**_ **pH 5.0** ^*b*^	**C = O/ aliphatics**	1542/2854	1.17	1.81	0.54	1.45	1.19
**AlCl**_**3**_ **pH 5.0** ^*b*^	**Carboxyl/ aliphatics**	1404/2923	1.02	1.18	0.37	1.17	1.33
**AlCl**_**3**_ **pH 5.0** ^*b*^	**Carboxyl/ aliphatics**	1404/2854	1.54	1.87	0.54	1.49	1.96
**AlCl**_**3**_ **pH 5.0** ^*b*^	**C-O /aliphatics**	1103/2923	2.73	3.99	3.48	7.10	11.44
**AlCl**_**3**_ **pH 5.0** ^*b*^	**C-O /aliphatics**	1103/2854	4.14	6.29	5.53	9.06	16.31
**PACl pH 5.0** ^*b*^	**Aromatics/aliphatics**	1630/2923	0.84	1.01	0.89	0.69	0.95
**PACl pH5.0** ^*b*^	**Aromatics/aliphatics**	1630/2854	1.40	1.53	1.24	0.88	1.50
**PACl pH5.0** ^*b*^	**C = O/ aliphatics**	1542/2923	0.53	0.78	0.34	0.33	0.42
**PACl pH 5.0** ^*b*^	**C = O/ aliphatics**	1542/2854	0.88	1.18	0.48	0.42	0.67
**PACl pH 5.0** ^*b*^	**Carboxyl/ aliphatics**	1404/2923	0.72	0.98	0.47	0.45	0.71
**PACl pH 5.0** ^*b*^	**Carboxyl/ aliphatics**	1404/2854	1.19	1.48	0.65	0.58	1.12
**PACl pH 5.0** ^*b*^	**C-O /aliphatics**	1103/2923	1.56	2.48	2.86	3.42	4.71
**PACl pH 5.0** ^*b*^	**C-O /aliphatics**	1103/2854	2.59	3.28	4.00	4.47	7.47
**PACl pH 6.0** ^*b*^	**Aromatics/aliphatics**	1630/2923	1.28	1.21	0.87	1.24	1.59
**PACl pH 6.0** ^*b*^	**Aromatics/aliphatics**	1630/2854	1.95	1.60	1.28	2.18	2.27
**PACl pH 6.0** ^*b*^	**C = O/ aliphatics**	1542/2923	1.10	1.23	0.46	1.21	0.23
**PACl pH 6.0** ^*b*^	**C = O/ aliphatics**	1542/2854	1.71	1.63	0.73	1.81	0.33
**PACl pH 6.0** ^*b*^	**Carboxyl/ aliphatics**	1404/2923	1.25	1.59	0.61	1.55	1.38
**PACl pH 6.0** ^*b*^	**Carboxyl/ aliphatics**	1404/2854	1.94	2.10	0.97	2.34	1.97
**PACl pH 6.0** ^*b*^	**C-O/ aliphatics**	1103/2923	3.25	3.91	3.84	8.02	25.56
**PACl pH 6.0** ^*b*^	**C-O /aliphatics**	1103/2854	5.04	5.92	5.61	12.31	37.61

^*a*^ FTIR data were obtained through calculating relative intensities for different peaks.

^*b*^ Coagulants dose = 0.8 mg Al/mg DOC.

The C = O stretching band at 1568 cm^−1^ [34] also shifted to lower frequency (1540–1550 cm^-1^), but intensity decreased to less. The peak at 1386 cm^-l^, which belonged to carboxylic or other oxygen-containing groups [33, 35], shifted to slightly higher frequency (1390–1410 cm^-l^) and increased in intensity. The 1404/2923, 1542/2923 and 1103/2923 ratios corresponded to carboxyl/aliphatics, C = O/aliphatics and C-O/aliphatic, respectively. Due to high ratios, flocs by PACl coagulation at pH 6.0 had more carboxylic structures, C = O and C-O groups than other flocs in all MW fractions. Accordingly, flocs by PACl coagulation at pH 5.0 with lower 1404/2923, 1542/2923 and 1103/2923 ratios had more aliphatics in all MW fractions. These were consistent with the results from 1404/2854, 1542/2854 and 1103/2854 ratios. Additionally, the differences between flocs at individual coagulation conditions did not related with MW obviously. Therefore, more aromatic structures were removed by AlCl_3_ coagulation at pH 5.0, while PACl coagulation at pH 6.0 and pH 5.0 removed more carboxylic structures and aliphatic structures, respectively. This agreed with previous research that Al_13_ species selectively bound to carboxylic groups at pH 6.0 [9].

### Characterization of composition on flocs surface by XPS analysis

**Fig 6:**
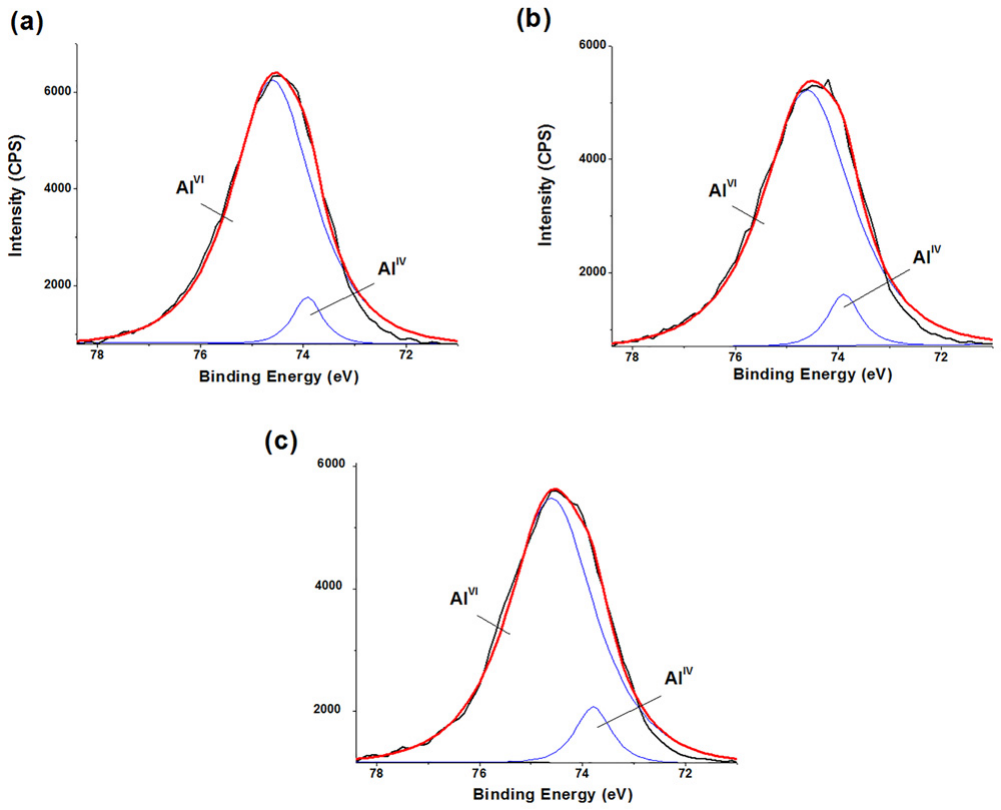
Al 2p XPS spectra of freeze-dried flocs by AlCl_3_ and PACl coagulation. (a) AlCl_3_, pH 5.0; (b) PACl, pH 5.0; (c) PACl, pH 6.0. Coagulants dose: 0.8 mg Al/mg DOC.

Based on the results mentioned above, we further identified the surface composition of coagulation flocs by XPS. Fig 6 shows the Al 2p XPS spectra of freeze-dried flocs coagulated by AlCl_3_ and PACl. Previous study indicated that tetrahedrally coordinated Al (Al^IV^, 73.7 eV) had a lower binding energy than octahedrally coordinated Al (Al^VI^, 74.2 eV) [36, 37]. In this study, the scans showed two overlapping bands associated with two different Al 2p transitions with binding energies of 73.9 eV and 74.6 eV, which correspond to Al^IV^ and Al^VI^, respectively. The Al^IV^/Al^VI^ ratio of flocs by AlCl_3_ coagulation at pH 5.0 was approximately 1:13.8 (Fig 6a), which clearly suggested that the flocs contained some amount of Al^IV^ center. This was in accordance with the results of Al speciation in flocs and solution that *in situ* formed Al_13_ were dominated aluminum species of AlCl_3_ at pH 5.0–5.5. [4, 6, 7, 38–40]. However, *in situ* Al_13_-HA complexes formed at pH 5.0 eventually decomposed into oligomeric Al-HA flocs during coagulation process, only a small amount of Al_13_ flocs were residual [7]. In this study, flocs still remained Al^IV^ centers, which may originate from *in situ* Al_13_ during coagulation. In the XPS spectrum of flocs by PACl coagulation at pH 5.0, the ratio of Al^IV^ and Al^VI^ was 1:12.1 (Fig 6b). Al^IV^/Al^VI^ ratio observed was very close to the theoretical value in Al_13_ molecules (1:12), where a central Al^IV^ is surrounded by 12 Al^VI^ [36, 37]. It implied that preformed Al_13_ species played dominated role in coagulation. However, on the surface of flocs by PACl coagulation at pH 6.0, observed Al^IV^/Al^VI^ ratio was approximately 1:11.4. Some researchers indicated that preformed Al_13_ polymers can form aggregated Al_13_ clusters at pH 6.0 and higher pH conditions [7, 41]. Lin et al. also found that some outer octahedral structures could decompose from Al_13_ molecules during the formation of Al_13_ aggregates [37]. In this study, due to decreased Al^VI^ during Al_13_ aggregation, the Al^VI^/Al^IV^ ratio was less than 12. Thus, it suggested that Al_13_ aggregates were in the flocs by PACl coagulation at pH 6.0. According to surface composition in Al flocs obtained from XPS analysis, predominant hydrolyzed Al species during coagulation can be concluded. For AlCl_3_, major species at pH 5.0 were *in situ* formed Al_13_. For PACl, preformed Al_13_ remained stable at pH 5.0, while aggregated Al_13_ clusters dominated at pH 6.0.

**Fig 7:**
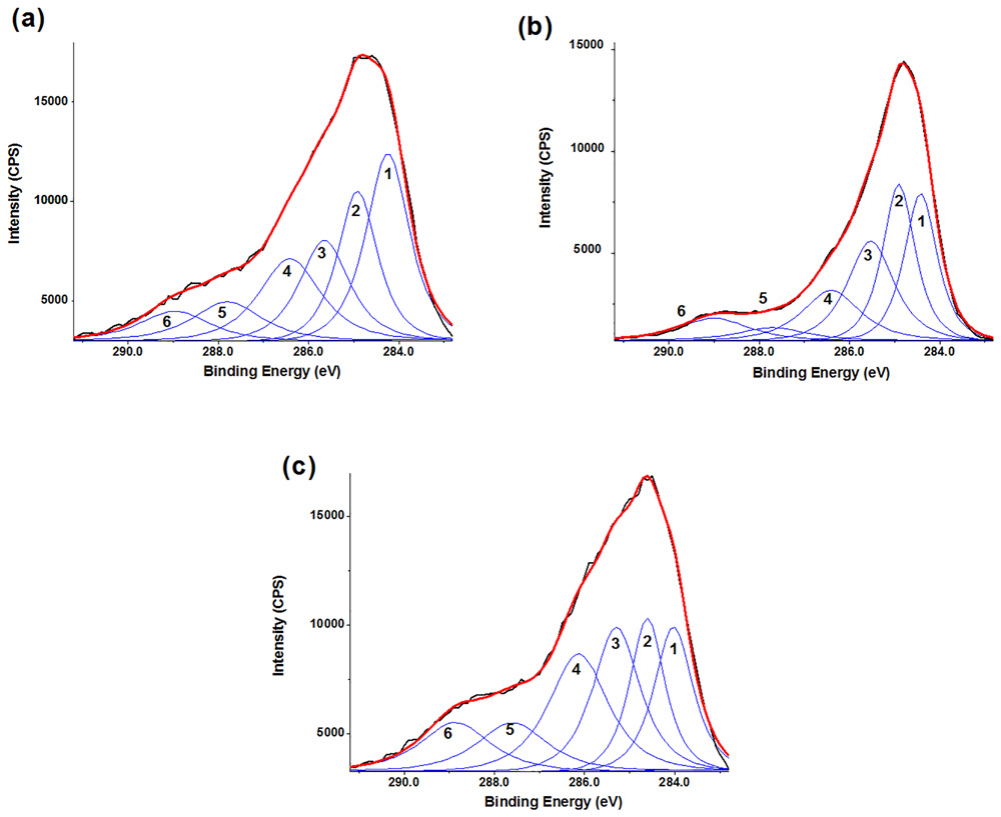
C 1s XPS spectra of freeze-dried flocs by AlCl_3_ and PACl coagulation. (a) AlCl_3_, pH 5.0; (b) PACl, pH 5.0; (c) PACl, pH 6.0. Coagulants dose: 0.8 mg Al/mg DOC, peak numbers correspond to the carbons mentioned in the text.

Background-subtracted C 1s XPS spectra of the flocs are shown in Fig 7, and the observed peaks are listed in Table 4. Six chemical states were fitted to the C 1s envelopes. Components associated with (1) unsubstituted aromatic carbon (C—C/C—H), (2) aliphatic carbon (C—C/C—H), (3) α-carbon (C—C(O)O), (4) ether or alcohol carbon (C—O), (5) ketonic carbon (C = O) and (6) carboxylic carbon (C(O)O) [42] were included in the three C 1s spectra. According to these assignments, aromatic carbon (28.1%) in the spectrum of flocs with AlCl_3_ at pH 5.0 (Fig 7a) was more than that of other flocs. Additionally, compared with other coagulation, flocs by PACl coagulation at pH 5.0 had more aliphatic carbon (25.7%), while flocs by PACl coagulation at pH 6.0 had more C-O (22.3%), C = O (10.7%) and C(O)O (carboxylic carbon, 10.8%). That is, AlCl_3_ coagulation removed more aromatic at pH 5.0. On the other hand, more aliphatic structures and carboxylic structures were removed by PACl coagulation at pH 5.0 and pH 6.0, respectively. These results were in accordance with the FTIR analysis mentioned above.

**Table 4:** Binding energies, full widths at half maximum (FWHM) and percent of total C for different chemical peaks in the C 1s XPS spectra (Fig 7).

Peak	AlCl_3_, pH 5.0			PACl, pH 5.0			PACl, pH 6.0		
	BE (eV)	FWHM (eV)	%	BE (eV)	FWHM (eV)	%	BE (eV)	FWHM (eV)	%
**Aromatic C-C/C-H**	284.3	1.13	28.1	284.4	0.85	24.3	284.0	1.00	17.7
**Aliphatic C-C/C-H**	284.9	1.03	20.6	284.9	0.84	25.7	284.6	0.90	16.9
**C-C(O)**	285.6	1.29	17.3	285.5	1.20	23.3	285.3	1.22	21.6
**C-O**	286.4	1.59	17.5	286.4	1.50	14.6	286.1	1.54	22.3
**C = O**	287.8	1.80	9.4	287.7	1.77	4.4	287.6	1.80	10.7
**C(O)O**	289.0	1.80	7.1	289.0	1.80	7.7	288.9	1.80	10.8

### Probing coagulation behavior of aluminum species for corresponding DBP precursors removal

On the basis of SUVA-DBPsFP correlations and FTIR, XPS results in this study, DBP precursors with corresponding characteristics were distinguished by MW and relations with SUVA values. Accordingly, coagulation behavior of aluminum species for DBP precursors differed from each other.

It can be indicated from the correlations of SUVA and TCAA (Figs 1–3) that main component of the TCAA precursors was aromatic structures, but THM-Br precursors had relatively low aromatic content. In particular, THM-Br precursors in high MW fractions had little aromatic structures. It agreed with the observation that THMs may have relatively more aliphatics as their precursors in addition to aromatic structures [1, 16]. For DCAA precursors and CHCl_3_ precursors, Observation from the SUVA-DBPsFP relations, clear differences among MW fractions can be found. On one hand, aromatic structures were important part for DCAA and CHCl_3_ precursors in low MW fractions (MW<30 kDa). On the other hand, the SUVA-DCAAFP relations and FTIR in MW>30 kDa fractions indicated that less aromatic structures in high MW fractions caused more contribution of other functional groups (e.g., carboxyl) on the DCAAFP. Furthermore, the FTIR results in this study suggested that high MW fractions contained more aliphatic structures.

According to the DBP precursors corresponding characteristics (MW and structure), the removals of DBP precursors by coagulation were distinguished. The greatest reduction of specific TCAAFP yields in all MW fractions occurred at pH 5.0 by AlCl_3_ coagulation (Table 1). FTIR and XPS results in this study also confirmed that aromatic structures were removed by AlCl_3_ coagulation at pH 5.0 to a more extent than other structures in all MW fractions. It agreed with the previous study [7] that more DBP precursors with aromatic and carboxylic structures were removed by AlCl_3_ than those by PACl at pH 5.0. TCAA precursors (relatively high aromatic content) regardless MW exhibited similar removal trends to that of aromatic structures.

Aluminum salts coagulation was not responsible for removing THM-Br precursors. Especially in high MW fractions, almost all THM-Br yields per DOC increased after coagulation, especially in high MW fractions (Table 1). The specific THMFP-Br yields reached minimum at pH 5.0 by PACl coagulation for all MW fractions. This agreed with FTIR results and XPS analysis that relatively more aliphatic structures were removed by PACl coagulation at pH 5.0. In contrast to average DOC values, THM-Br still had aliphatic structures as their precursors in all MW fractions. Thus, accordingly to the minimum THMFP-Br yields after coagulation, PACl at pH 5.0 were more responsible for coagulating THM-Br precursors with aliphatic structures and relatively low aromatic content without MW limits.

For DCAA precursors and CHCl_3_ precursors, in low MW fractions (MW<30 kDa), the greatest removal for both DCAA and CHCl_3_ precursors occurred at pH 5.0 by AlCl_3_ coagulation (Table 1), which resembled the best removal of TCAA precursors. Furthermore, the greatest removal of DCAA precursors in high MW fractions (MW>30 kDa) occurred at pH 6.0 by PACl coagulation (Table 1). It was consistent with the FTIR and XPS results that more carboxylic structures could be removed by PACl coagulation at pH 6.0. Accordingly, DCAA precursors with MW>30 kDa had relatively more carboxylic structures as their precursors. Thus, more these DBP precursors were removed by PACl coagulation at pH 6.0. In contrast to DCAA precursors, the greatest removal of CHCl_3_ precursors with MW>30 kDa were by PACl coagulation at pH 5.0 (Table 1). These results were consistent with the FTIR and XPS results that more aliphatic structures could be removed by PACl coagulation at pH 5.0. Therefore, similar to THM-Br precursors, CHCl_3_ precursors in MW>30 kDa fractions with aliphatics and relatively low aromatic structures were removed preferentially by PACl coagulation at pH 5.0.

## Conclusion

1. SUVA is a good and simple surrogate for aromatic content of DBPFP, and the effect of SUVA to DBPsFP was variable by different MWs. There was a significant correlation between SUVA and TCAAFP yields in all different MW fractions (*R* = 0.710, 0.777, 0.824 and 0.828, respectively. *p* < 0.05). The SUVA values showed negative correlations with the yields of THMFP-Br in MW>100 and 30-100k Da fractions (*R* = -0.645 and -0.767, respectively. *p* < 0.05). The correlations between SUVA and DCAAFP and CHCl_3_FP yields showed significant correlations in low MW fractions, but not significant in high MW fractions.
2. The aromaticity and characteristics of DBP precursors can be classified by the relations with SUVA values as follows: TCAA precursors (no MW limits), DCAA and CHCl_3_ precursors in low MW fractions (MW<30 kDa), had relatively high aromatic content; THM-Br precursors (no MW limits) and CHCl_3_ precursors in high MW fractions (MW>30 kDa) had relatively low aromatic content and more aliphatics structures; DCAA precursors in high MW fractions (MW>30 kDa) had relatively low aromatic content and more carboxylic structures.
3. For DBP precursors with high aromatic content, AlCl_3_ coagulation at pH 5.0 removed more TCAAFP, DCAA precursors (MW<30 kDa) and CHCl_3_ precursors (MW<30 kDa). More DCAA precursors (MW>30 kDa) with relatively low aromatic content and more carboxylic structures were removed by PACl coagulation at pH 6.0. For DBP precursors with aliphatics and relatively low aromatic structures, THM-Br precursors and CHCl_3_ precursors (MW>30 kDa) were preferentially.

## Supporting Information

S1 File S1 Table: Correlation matrix of SUVA index and four DBPsFP in different MW fractions. S1 Fig: Property—property plots of PCA factor loadings between SUVA indexand four DBPsFP in different MW fractions. (RAR)
